# Productivity growth of skilled nursing facilities in the treatment of post-acute-care-intensive conditions

**DOI:** 10.1371/journal.pone.0215876

**Published:** 2019-04-19

**Authors:** Jing Gu, Neeraj Sood, Abe Dunn, John Romley

**Affiliations:** 1 School of Pharmacy, University of Southern California, Los Angeles, California, United States of America; 2 Sol Price School of Public Policy, University of Southern California, Los Angeles, California, United States of America; 3 U.S. Bureau of Economic Analysis, Washington D.C., United States of America; Medical University Graz, AUSTRIA

## Abstract

**Background:**

Health care is believed to be suffered from a “cost disease,” in which a heavy reliance on labor limits opportunities for efficiencies stemming from technological improvement. Although recent evidence shows that U.S. hospitals have experienced a positive trend of productivity growth, skilled nursing facilities are relatively “low-tech” compared to hospitals, leading some to worry that productivity at skilled nursing facilities will lag behind the rest of the economy.

**Objective:**

To assess productivity growth among skilled nursing facilities (SNFs) in the treatment of conditions which frequently involve substantial post-acute care after hospital discharge.

**Methods:**

We constructed an analytic file with the records of Medicare beneficiaries that were discharged from acute-care hospitals to SNFs with stroke, hip fracture, or lower extremity joint replacement (LEJR) between 2006 and 2014. We populated each record for 90 days starting at the time of SNF admission, detailing for each day the treatment site and all associated costs. We used ordinary least square regression to estimate growth in SNF productivity, measured by the ratio of “high-quality SNF stays” to total treatment costs. The primary definition of a high-quality stay was a stay that ended with the return of the patient to the community within 90 days after SNF admission. We controlled for patient demographics and comorbidities in the regression analyses.

**Results:**

Our sample included 1,076,066 patient stays at 14,394 SNFs with LEJR, 315,546 patient stays at 14,154 SNFs with stroke, and 739,608 patient stays at 14,588 SNFs with hip fracture. SNFs improved their productivity in the treatment of patients with LEJR, stroke, and hip fracture by 1.1%, 2.2%, and 2.0% per year, respectively. That pattern was robust to a number of alternative specifications. Regressions on year dummies showed that the productivity first decreased and then increased, with a lowest point in 2011. Over the study period, quality continued to rise, but dominated by higher costs at first. Costs then started to decrease, driving productivity to grow.

**Conclusion:**

There has been substantial productivity growth in recent years among SNFs in the U.S. in the treatment of post-acute-care-intensive conditions.

## Introduction

Health care is believed to suffer from a “cost disease,” which means that the costs rise at a rate significantly greater than the rate of inflation, because the quantity of labor required to produce health care services is difficult to reduce [1]. Although new technologies that aim to increase efficiency have been introduced into health care, they have done little to lower costs [2]. As a result, there is wide interest in increasing the productivity of the U.S. health care system. According to the Institute of Medicine, “the only sensible way to restrain costs is to enhance the value of the health care system, thus extracting more benefit from the dollars spent” [3].

The Bureau of Labor Statistics defines multifactor productivity as “output per unit of labor, capital, and other measurable inputs,” which reflects “intangible influences…such as improvements in efficiency and technology.”[4] There is recent evidence of a positive trend in hospitals’ multifactor productivity. A study from the Centers for Medicare and Medicaid Services (CMS) showed that the most recent 10-year moving-average growth rate of hospitals’ multifactor productivity (MFP), ending in 2013, was 0.1–0.5% [5]. Using Medicare data from 2002 to 2011, Romley et al. found that the rate of annual quality-adjusted MFP growth among hospitals was 0.8%, 0.6%, and 1.9% in the treatment of patients with heart attack, heart failure, and pneumonia, respectively [6].

By contrast, the Bureau of Labor Statistics reported that MFP for hospitals together with nursing and residential care facilities decreased by 0.4% annually from 2006 to 2014 [7], which may indicate that nursing homes are not sharing the same productivity growth that hospitals have shown. In addition, nursing homes are relatively “low-tech” compared with hospitals, making them theoretically more vulnerable to cost disease [8].

Skilled nursing facilities (SNFs) are a particular type of nursing home that provide short-term, skilled nursing care and rehabilitation services, such as physical and occupational therapy and speech-language pathology services, to patients following a stay in an acute-care hospital [9]. SNF services are covered by Medicare, so detailed administrative data are available for the study of SNF performance. According to the National Health Expenditure Accounts, Medicare is the second largest payer for nursing home care, paying 23% of the total in 2016 [10].

There is wide concern about Medicare’s increasing expenditures on SNFs and its payment system. Medicare payments for post-acute care have grown faster than most other categories of spending, and SNFs account for over 50% of the total Medicare expenditures on post-acute care [11]. There is concern that the current prospective payment system to pay SNFs for each day of service might induce SNFs to keep patients longer than necessary and/or furnish therapy services that are unrelated to a given patient’s condition, which threatens the quality of care and the productivity of the SNFs [9, 12]. The Medicare payment policy also results in highly fragmented health care delivery [9]. Under the payment policy, acute-care hospitals and SNFs each receive a separate payment for providing acute and post-acute care, and the policy does not reimburse any entity for coordinating patient transitions across providers [13]. The lack of coordination and fragmentation of care potentially lower productivity. In response to those concerns, the Affordable Care Act included several Medicare reforms such as penalties for readmissions, “Accountable Care Organizations,” and “Bundled Payments” to improve care coordination, particularly after hospital discharge [14]. Since then, there has been an increase in “virtual” integration between hospitals and SNFs, meaning that some hospitals started to form a “preferred” SNF network and to discharge most of their patients to SNFs within their network [15]. Studies have shown that such integration potentially lowers costs and improves quality of care, resulting in higher productivity [16–18]. It is unclear, however, how productivity growth among SNFs has changed over the years and whether recent Medicare payment reforms emphasizing care coordination have made a difference.

Few studies have examined MFP at SNFs. A study of residential care facilities in Canada found that *labor* productivity (which is influenced by MFP) was virtually unchanged from 1984 to 2009 [19]. However, an important limitation of that study is that it did not account for differences in the quality of care received by patients during SNF stays. Productivity might appear to be decreasing over time if SNFs are treating more severely ill patients, providing better quality of care, or both [6]. In quantifying trends in SNF productivity, it is important to address all such potentially confounding factors. To our knowledge, no study has directly reported on the quality-adjusted productivity of SNFs in the U.S. This study is intended to fill the research gap by analyzing quality-adjusted productivity for SNF stays involving the most common conditions in post-acute-care settings.

## Materials and methods

### Population

We analyzed data from Medicare fee-for-service beneficiaries discharged to SNFs from short-term acute-care hospitals from 2006 to 2014. We restricted our analysis to SNF stays that started within 90 days of hospital discharge and were the first post-acute care (including care received in Home Health Agencies, or HHA) that the beneficiary received after being discharged from the hospital. We identified patients with lower extremity joint replacement (LEJR), hip fracture, or stroke according to the principal diagnosis code used during hospitalization [20]. Those three conditions are common in the elderly population of the U.S. and result in high usage of post-acute care [21]. We used Medicare Provider Analysis and Review (MedPAR) files as our primary data source [22]. The University of Southern California Institutional Review Boards has approved this study. The form of consent was not obtained because the data were analyzed anonymously.

### Production function framework

MFP is defined as a measure of economic performance that compares the amount of goods and services produced (output) to the amount of combined resources (inputs) used to produce those goods and services [23, 24]. The inputs may include labor, capital, energy, materials, and purchased services [7]. We characterized the output as the number of “high-quality” stays produced by a SNF for patients with a condition in a year. We summarized the inputs as the total treatment costs of a SNF in treating a condition in a year. It is important to consider control variables such as the severity of illness because the outcomes and the amount of resources needed to achieve them are likely to depend on the initial condition of the patient. Building on our prior work on hospitals, we applied a productivity framework to SNFs that uses the logarithm of the ratio of the output to the input as the dependent variable [6].

### Measures

#### Output

We characterized the output in the production function as the number of high-quality SNF stays. A high-quality stay was one in which the patient was alive and had returned to the community (with or without HHA care) as of 90 days after SNF admission. We obtained information about mortality from Master Beneficiary Summary files. We determined whether patients were residing in the community (potentially receiving HHA services at home) using combined information from MedPAR, the Minimum Data Set (MDS), and Home Health Standard Analytic Files [21, 25, 26]. The MDS contains assessments of health status for all residents of nursing homes certified by Medicare or Medicaid. We applied an algorithm to group the assessments into stays linked with our sample (S1 Text), which enabled us to ascertain readmissions to facilities and residence in facilities as of day 90 after SNF admission, including custodial nursing homes financed by Medicaid.

We also considered two alternative definitions for high-quality stays. The first alternative defined high-quality stays as stays where patients were discharged from hospitals directly to home or HHA, as indicated by the discharge destination codes from Medicare claims. The second alternative definition was the strictest one. It defined high-quality stays as stays where the patient continuously resided in the community for 30 days without any readmission during a 90-day window.

We assessed the outcomes using a 90-day window after SNF admission. To test whether the length of the window would influence our results, we performed three sensitivity analyses using a 30-day window, a 60-day window and a 120-day window, respectively.

#### Inputs

We defined the input as the total costs incurred during SNF stays. We converted Medicare payments to costs using cost-to-revenue ratios that SNFs submit as part of their cost accounting reports to the CMS [21, 25]. This input measure accounts for the opportunity cost to society of employing scarce resources for treatment [6]. We also performed a supplementary analysis from the payer perspective using Medicare payments. We adjusted all costs and payments for inflation to 2014 dollars using market basket indices from the CMS [27].

#### Controls

We obtained information about patient characteristics such as age, sex, race/ethnicity, and Medicare enrollment from the Master Beneficiary Summary files. Elixhauser comorbidities were based on hospitalization claims [28] and constructed from the first 10 diagnosis codes of the index hospitalization.

In sensitivity analysis, we added the length of hospital stay and the number of days between hospital discharge and SNF admission as control variables, as they may indicate the severity of illness. In a separate sensitivity analysis, we followed the calculation method for inpatient quality indicators published by the Agency for Healthcare Research and Quality (AHRQ) and included the risk-adjusted mortality rate for each condition in order to separate the quality produced by the hospitals from that produced by the SNFs [29].

To determine whether vertical integration between hospitals and SNFs has an impact on productivity growth, we constructed an indicator of integration for each SNF using the percentage of admitted patients who were discharged from the SNF’s parent hospital. We used the variable “related provider number” from Medicare Provider of Services (POS) files to identify each SNF’s parent hospital [30].

### Analysis

In order to study productivity growth and how adjustment for quality affects the trend, we first looked at unadjusted productivity growth by defining output as the number of patient stays. Productivity was measured as the ratio of output to inputs. For each condition at each SNF in each year, we regressed the logarithm of unadjusted productivity on the trend variable (year) using the Ordinary Least Square (OLS) model, in order to get the compound growth rate from 2006 through 2014, as shown in Eq (1). Next, we performed the same regressions but included patient characteristics and severity of illness as control variables, as shown in Eq (2), where *X* is a vector for all control variables. Finally, we incorporated quality of care by defining the output as high-quality stays in which the patient was alive and had returned to the community within 90 days after SNF admission, as shown in Eq (3) below. All regressions were weighted by the number of patients at each SNF.

$$\log\left(unadjusted\;productivity\right)=\beta_{1}year$$ (1)

$$\log\left(unadjusted\;productivity\right)=\beta_{1}year+{X\beta }_{2}$$ (2)

$$\log\left(quality\;adjusted\;productivity\right)=\beta_{1}year+{X\beta }_{2}$$ (3)

We also regressed costs, payments, and the rate of return of patients to the community on the trend variable separately (S2 Text). In addition, we analyzed productivity growth in each year by replacing the single trend variable with binary variables for each year. As a supplemental analysis, we added an interaction term between the vertical integration rate and the trend variable to study whether vertical integration has an effect on productivity growth.

## Results

Our sample included 1,076,066 patient stays with a diagnosis of LEJR at 14,394 SNFs, 315,546 patient stays with a diagnosis of stroke at 14,154 SNFs, and 739,608 patient stays with a diagnosis of hip fracture at 14,588 SNFs.

Table 1 shows a summary of the patient characteristics and outcomes. The average age of the patients in our sample ranged from 79 years for LEJR to 84 for hip fracture. Each patient had 2–3 comorbid conditions on average. The average length of hospital stay was 5–6 days, with less than 1 day between hospital discharge and SNF admission. The inpatient risk-adjusted mortality rate was 0.1% for hip replacement, 3.1% for hip fracture and 8.0% for stroke. The rate of survival and return to community was 77%, 56%, and 57% for LEJR, stroke, and hip fracture, respectively. The amount of Medicare payments for SNF care ranged from $11,441 to $15,679, and the corresponding costs ranged from $9,497 to $12,993.

**Table 1:** Summary statistics of patient characteristics and outcomes.

	Lower Extremity Joint Replacement	Stroke	Hip Fracture
	N = 80,480 SNF-years	N = 52,790 SNF-years	N = 76,570 SNF-years
**Age**	79.4 (4.4)	82.6 (4.9)	84.2 (4.0)
**Male (%)**	26.8 (24.3)	33.9 (28.9)	24.5 (21.8)
**Race/ethnicity**			
**White (%)**	90.1 (21.0)	85.0 (26.2)	92.3 (17.8)
**Black (%)**	6.0 (16.7)	10.6 (22.6)	3.9 (13.1)
**Other (%)**	3.9 (13.9)	4.4 (14.6)	3.7 (12.4)
**Elixhauser comorbidity counts**	2.4 (0.7)	2.4 (0.8)	2.6 (0.7)
**Hospital length of stay**	5.0 (2.0)	6.4 (3.1)	5.9 (2.0)
**Days between hospital discharge and SNF admission**	0.3 (1.9)	0.7 (3.1)	0.5 (2.4)
**Inpatient risk adjusted mortality rate (%)**	0.1 (2.1)	8.0 (5.6)	3.1 (4.2)
**Alive and residing in community in 90 days post SNF admission (%)**	78.1 (21.4)	56.9 (26.6)	58.0 (23.9)
**SNF costs (2014$)**	9,497 (5,294)	11,159 (6,494)	12,993 (6,279)
**SNF Medicare payments (2014$)**	11,441 (5,722)	13,586 (7,077)	15,679 (6,606)

Standard deviations are in parentheses. SNF costs and Medicare payments are average costs and payments per SNF per year, unweighted for the number of patients received per SNF per year, adjusted for inflation to 2014 dollars using market basket indices from the CMS.

**Fig 1:**
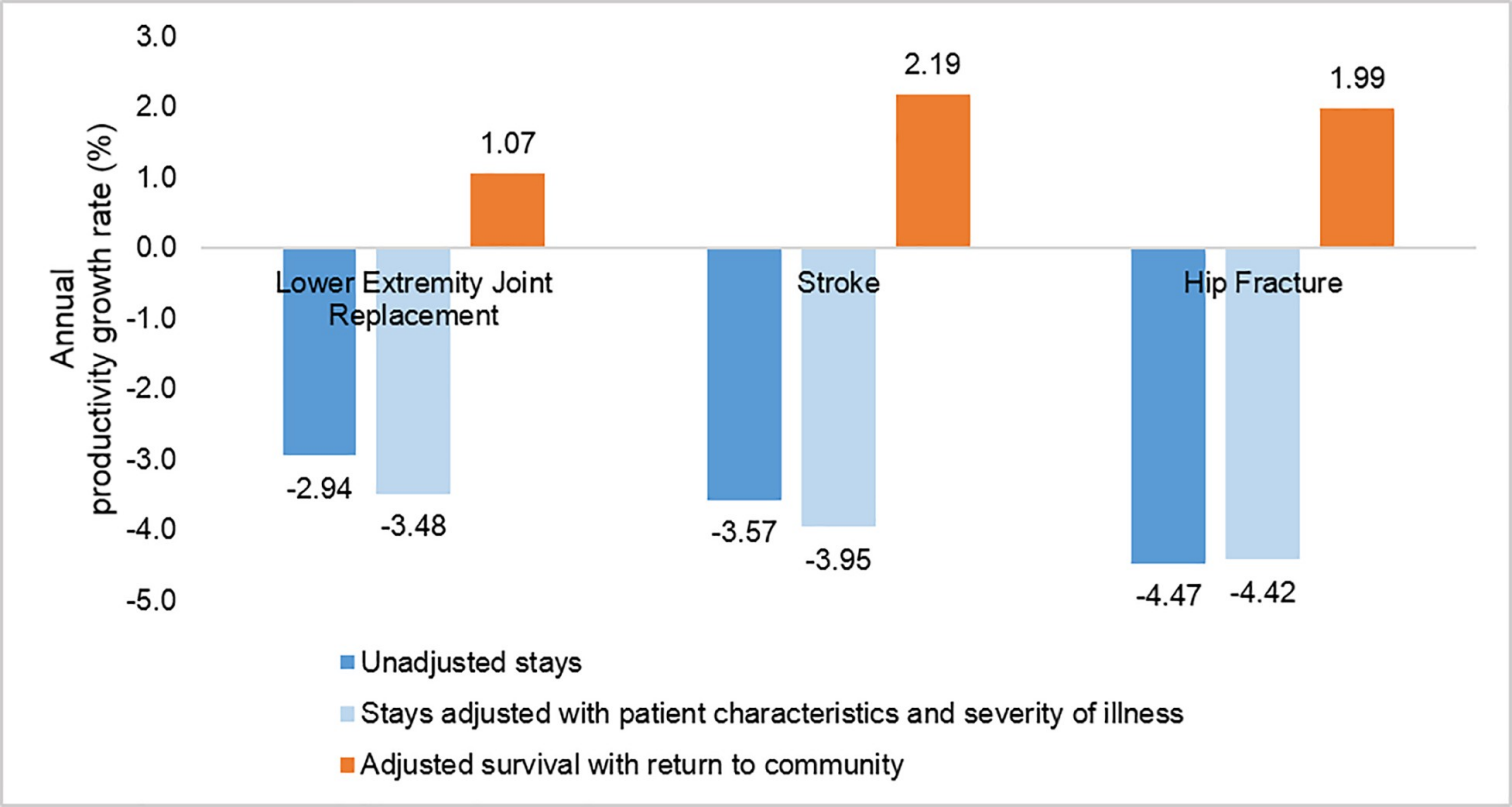
Unadjusted and adjusted annual rates of SNF productivity growth for three conditions, 2006–2014. Note. All rates are significantly different from zero (p<0.05).

The growth rate of unadjusted productivity (without controlling for patient characteristics and severity of illness) was negative for all three conditions (Fig 1). The negative growth estimates mainly resulted from increasing treatment costs from 2006 to 2014. After we controlled for patient characteristics and the severity of illness, productivity growth was still negative, and the annual rate did not change substantially compared to the unadjusted rate. However, productivity for LEJR and stroke decreased somewhat more with severity adjustment.

When we controlled for patient characteristics and comorbidities and defined the output as stays where the patient survived and was residing in the community 90 days after SNF admission, the annual rate of productivity growth became positive for all three conditions. The productivity growth was due to improvement over time in the quality of care as measured by survival and return to the community.

**Fig 2:**
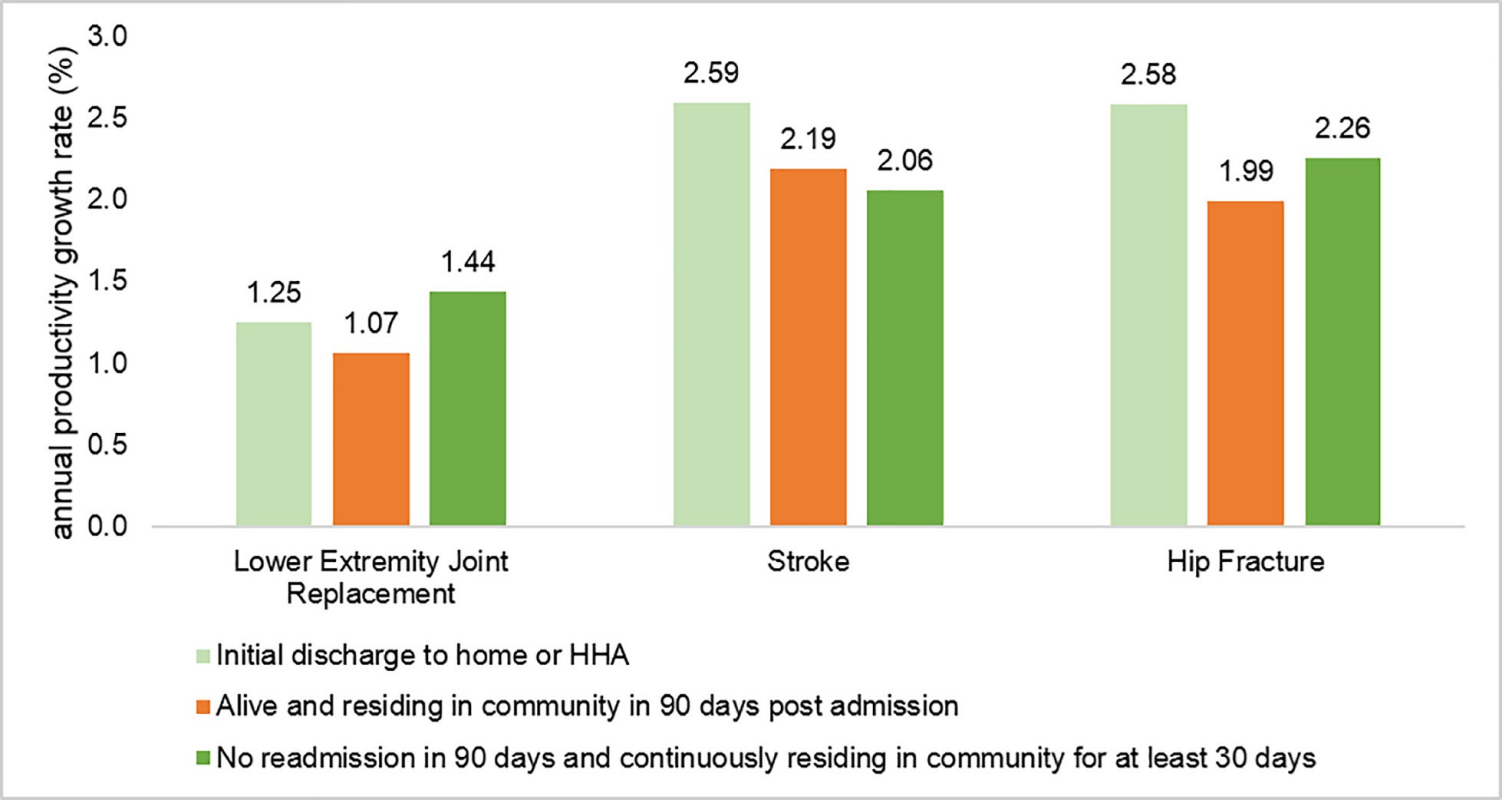
Annual rates of SNF productivity growth for three conditions, based on alternative definitions of quality, 2006–2014. Note. All rates are significantly different from zero (p<0.05).

We had two alternative definitions of a high-quality SNF stay. As shown in Fig 2, there was growth in productivity over the study period for all three conditions no matter which definition of high-quality stay we used.

**Fig 3:**
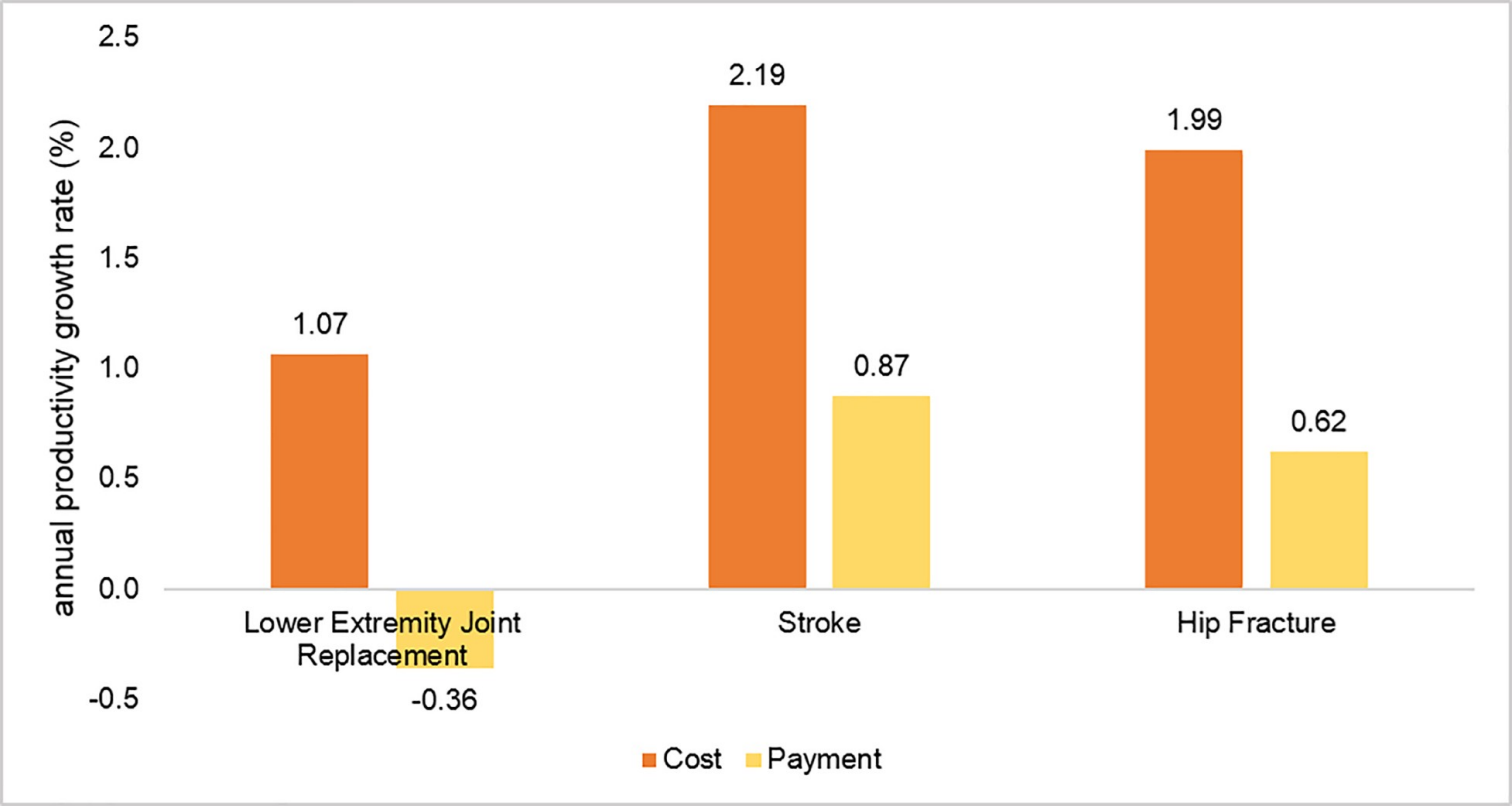
Annual rates of SNF productivity growth for three conditions based on costs vs. those based on payments, 2006–2014. Note. All rates are significantly different from zero (p<0.05).

Fig 3 shows a comparison of the productivity growth rate for each condition calculated on the basis of costs versus that calculated on the basis of Medicare payments. The productivity growth based on costs was higher than the productivity growth based on Medicare payments for all three conditions. For LEJR, the productivity growth was negative when the input was measured using Medicare payments. When we changed the 90-day window to 60 days or 120 days, the productivity growth rate was still positive for all three conditions (S1 Fig).

**Fig 4:**
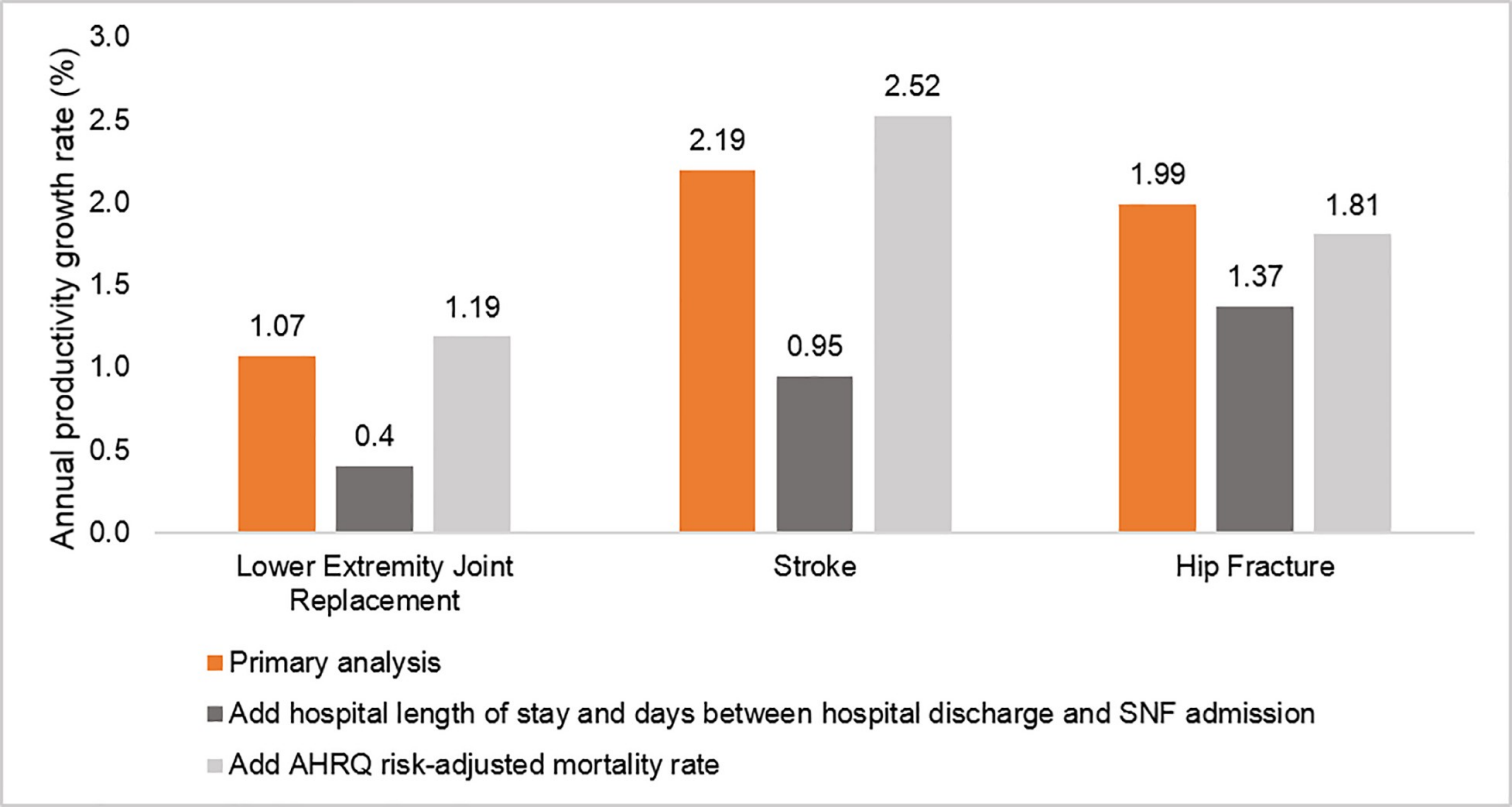
Annual rates of SNF productivity growth for three conditions, sensitivity analysis results, 2006–2014. Note. All rates are significantly different from zero (p<0.05).

For sensitivity analyses, we performed regressions using several covariates. When we added the length of hospital stay and the number of days between hospital discharge and SNF admission as two additional control variables, the productivity growth rates decreased by roughly half but were still significantly positive. When we included the inpatient risk-adjusted mortality rate, the productivity growth was similar to our primary specification (Fig 4).

**Fig 5:**
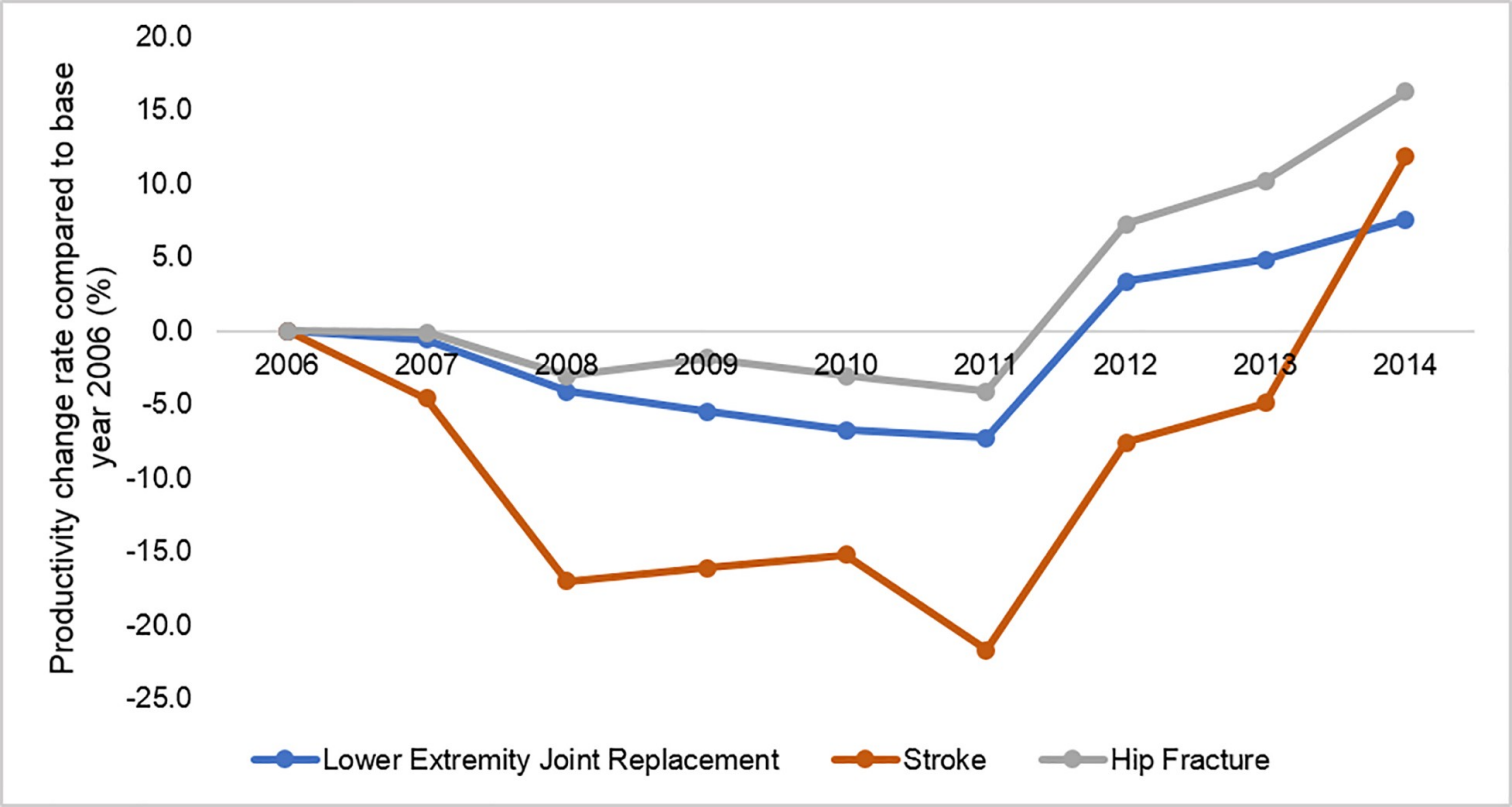
Cumulative productivity change for three conditions, 2006–2014.

We also regressed the logarithm of the productivity on year dummies and all other covariates to show the change in productivity in each year from 2007 to 2014 compared with the base year 2006. The productivity mostly decreased until 2011 and then increased through 2014 (Fig 5). That trend was mainly driven by changes in costs. Over the study period, the rate of high-quality SNF stays continued to rise, but the productivity was dominated by increasing costs at first. Later on, the costs started to decrease, resulting in productivity growth overall.

SNFs with higher degree of vertical integration had faster productivity growth in the treatment of LEJR; however, vertical integration had no significant effect on the rate of productivity growth in the treatment of the other two conditions (Table 2). For all three conditions, productivity was positively correlated with the degree of integration. The rate of vertical integration decreased annually from 2006 to 2014 (S2 Fig), which indicates that SNFs with higher productivity were crowded out of the market. Since 2013, the integration rate increased slightly, except for the stroke condition.

**Table 2:** Estimated percent of productivity growth and interaction with vertical integration.

	Lower Extremity Joint Replacement	Stroke	Hip Fracture
**Year**	2.01** (0.09)	3.17** (0.17)	2.57** (0.10)
**Integrated**	1.14** (0.01)	1.42** (0.02)	1.33** (0.02)
**Year × Integrated**	0.82** (0.23)	-0.59 (0.51)	-0.43 (0.34)

Standard errors in parentheses.

* p<0.05

** p<0.01

*** p<0.001

## Discussion

We assessed productivity growth from 2006 to 2014 among SNFs treating Medicare beneficiaries who had been admitted to hospitals with common conditions that frequently involve post-acute care. For all three conditions, the unadjusted productivity growth was negative. However, after adjusting for disease severity and quality of care, we found substantial productivity growth ranging from 1.1% to 2.2% per year. The results were robust across several sensitivity analyses.

An understanding of productivity trends is important, as the Affordable Care Act now requires that the market basket percentage under the Medicare Prospective Payment System be reduced annually by a productivity adjustment [5]. A rationale for the adjustment is that Medicare should benefit from productivity gains in the economy at large. However, if productivity grows relatively rapidly in the broader economy, productivity gains among providers might not be sufficient to offset slower growth in reimbursement and resources under the Affordable Care Act, and providers might struggle to maintain quality of care [6]. Our prior results suggest that such concerns might be overstated, at least in the inpatient setting [6].

However, this finding would not necessarily have generalized to the post-acute care setting. Studies have shown that post-acute care is the largest driver of the geographic variation in Medicare spending, and there is much uncertainty about the clinical appropriateness of post-acute care sites for particular patients [9, 31–33]. The Bureau of Labor Statistics reported a negative rate of productivity growth for hospitals plus nursing and residential care facilities [7]. That report did not account for variation in disease severity and quality of care, however. We addressed those factors by using “high-quality stays” as the output of SNFs, and also tried to separate the quality of care produced by acute-care hospitals from that produced by SNFs. The definition of “high-quality stays”–survival and return to community as of 90 days after SNF admission–was motivated by CMS policies with respect to public reporting and reimbursement. For example, CMS and Hospital Quality Alliance (HQA) publicly report 30-day mortality measures for certain conditions since 2007 as an important indicator for quality of care [34]. In terms of skilled nursing facilities, CMS has the SNF Quality Reporting Program (QRP) that publicly reports SNF provider performance on the quality measures, and one of the five measures is the rate of successful return to home or community from an SNF [35]. The outcomes analyzed here are widely studied in health services and health policy [36–39]. Romley et al. focused on hospital stays, and therefore considered mortality and readmissions [6]. This study also considers return to community, because it is an important outcome in post-acute care [25, 40–42].

We also considered two alternative definitions for high-quality stays. The first alternative defined high-quality stays as stays where patients were discharged from hospitals directly to home or HHA, as indicated by the discharge destination codes from Medicare claims. That was the approach used in the Improving Medicare Post-Acute Care Transformation (IMPACT) Act of 2014, which required post-acute care providers to report such information as a quality metric [43]. That approach fails, however, to capture readmissions to facilities and residence. The second alternative definition was the strictest one. It defined high-quality stays as stays where the patient continuously resided in the community for 30 days without any readmission during a 90-day window. We used 90 days as the primary window because of several considerations. Firstly, 90 days is a common length of episode used in many published studies about post-acute care [21, 44, 45]. Some studies used even longer window, such as 120 days [25]. Secondly, SNF patients typically need a longer time to recover and the length of stays are usually longer than hospital patients. Thus we used a longer window to look at SNF patients’ outcomes than 30 days, which is commonly used in studies on hospitals. We do recognize that there’s no perfect standard about the length of episodes when studying outcomes among SNF patients. Therefore, we did sensitivity analysis using different length of windows to define outcomes, including 30-day, 60-day, 90-day and 120-day, in order to show robustness.

Our results do not support the hypothesis that SNFs suffer from a cost disease. That suggests that similar to hospitals, SNFs have had positive quality-adjusted productivity growth in recent years. Thus, the concerns about a decline of quality of care when reimbursement does not keep up with health care cost inflation may be overstated in post-acute care setting, too. Conventional measures of health care prices tend to ignore quality of care and overstate true inflation [6, 46]. Moreover, innovative payment and delivery approaches under the ACA and among private payers may increase the incentives for SNFs to achieve productivity gains in the future. In addition, we found that vertically integrated SNFs are more productive than non-integrated SNFs. If there were no decline in the percentage of vertically integrated SNFs in recent years, we might have observed even greater productivity growth among the SNFs. Our results indicate that there is a need for some reassessment of the performance of the U.S. health care system and for payment reforms emphasizing integration and coordination between hospitals and post-acute care facilities.

Our study has several limitations. Quality is complex and difficult to measure. MDS files lack discharge dates for some nursing home stays, which causes some measurement error. To address that issue, we defined quality in three different ways. All three definitions of quality led to similar results. We also performed analyses using different follow-up periods and found similar productivity growth for three different time windows. As in our prior work, we focused on the site of care, instead of the full episode, which gives a more comprehensive measure of health care output. Productivity improvement might be better or worse if it is measured in terms of episodes rather than sites of care. That is an important direction for future research.

In conclusion, our results suggest that similar to hospitals, SNFs have not suffered from what has been called a cost disease, in which technological change does not generate efficiencies to offset increasingly costly labor. Much work remains to be done on this important topic.

## Supporting information

S1 Fig Annual rates of SNF productivity growth for three conditions, based on return to community rate in 30, 60, 90 (baseline) vs. 120 days, 2006–2014. Note. All rates are significantly different from zero (p<0.05).
(TIF)

S2 Fig Vertical integration rate for three conditions, 2006–2014. (TIF)

S1 Text Algorithm to Process the MDS Data. (DOCX)

S2 Text Changes in Output and Input over Years. (DOCX)
