# Supplementary figures and images for: Productivity growth of skilled nursing facilities in the treatment of post-acute-care-intensive conditions
Source: PLoS One. 2019 Apr 19;14(4):e0215876. doi: 10.1371/journal.pone.0215876 (PMC6474610; doi:10.1371/journal.pone.0215876)

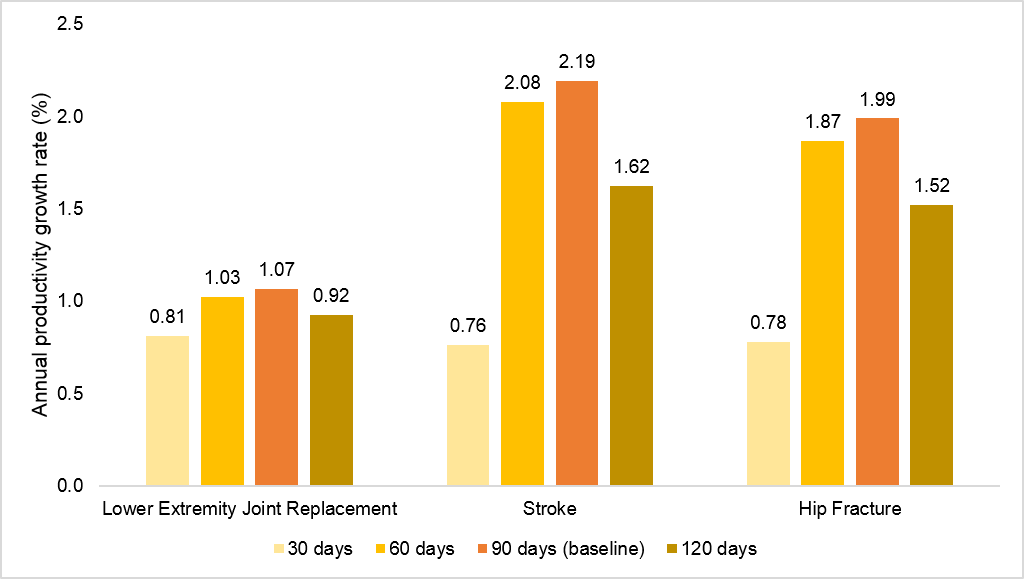

Supplement: S1 Fig — Note. All rates are significantly different from zero (p<0.05). (TIF) [file pone.0215876.s001.tif]

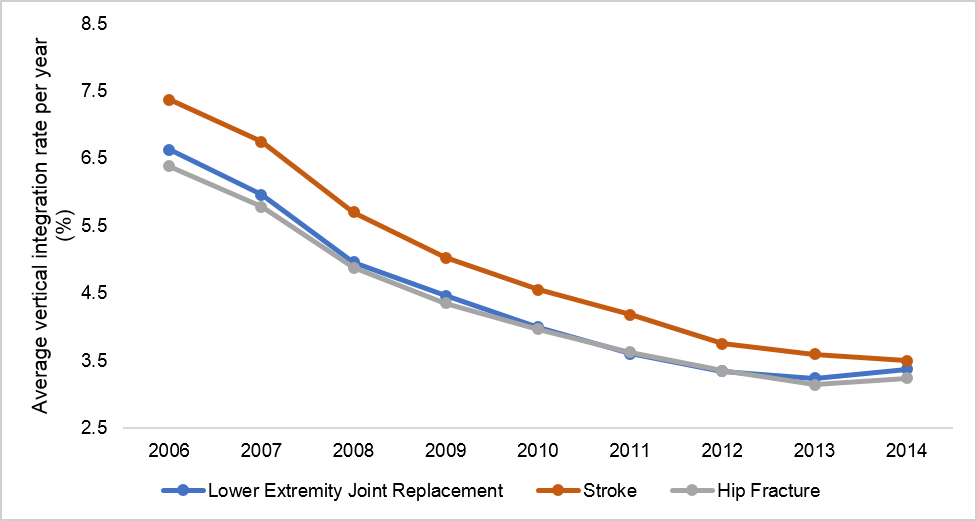

Supplement: S2 Fig — (TIF) [file pone.0215876.s002.tif]
