# Supplementary material for: Productivity growth of skilled nursing facilities in the treatment of post-acute-care-intensive conditions
Source: PLoS One. 2019 Apr 19;14(4):e0215876. doi: 10.1371/journal.pone.0215876 (PMC6474610; doi:10.1371/journal.pone.0215876)
Supplement: S1 Text — (DOCX) [file pone.0215876.s003.docx]

**S1 Text. Algorithm to Process the MDS Data**

We used an algorithm to group assessment-level MDS data into stay-level data. First, we combined all of the years of the raw MDS data from 2006 to 2014. Then we sorted the dataset by Beneficiary ID and Assessment Date so that we could see the beneficiaries’ chronological progression through various nursing homes. We designated the first observation for each beneficiary as the starting point for the first stay. Then, we moved on to the next observation. If there was a discharge date, we flagged that as the last observation in the stay and designated the next observation for the same beneficiary as the first assessment of the second stay. If the discharge date was missing, we considered each observation after the first as an intermediate assessment within the same stay. We continued until either we found a discharge date or a new beneficiary appeared in the file.

In the event that there were no discharge dates, we assumed that the beneficiary had a single stay and was still in the nursing home as of the last day of our data.

The dataset changed from MDS2.0 to MDS3.0 in 2010. To smooth the MDS2.0 and MDS3.0 data together, we started by appending the two stay-level files together and sorted by beneficiary ID and entry date. Presumably, if the MDS2.0 stay flows into the MDS3.0 file, there should be one stay with an entry date before or on 9/30/2010 and a discharge date (which was imputed) on 9/30/2010. There should then be a stay in the next observation with an entry date occurring before 9/30/2010 and a discharge date after 9/30/2010. If those conditions were met, we kept the earliest Entry Date between the two adjacent stays and the latest Discharge Date between the two adjacent stays. We then had two identical stays by beneficiary ID, entry date, and discharge date, so we eliminated the duplicate stays on the basis of those variables.
