# Supplementary material for: Productivity growth of skilled nursing facilities in the treatment of post-acute-care-intensive conditions
Source: PLoS One. 2019 Apr 19;14(4):e0215876. doi: 10.1371/journal.pone.0215876 (PMC6474610; doi:10.1371/journal.pone.0215876)

S2 Text. Changes in Output and Input over Years

As shown in S2 Figure a and S2 Figure b, the output measure (rate of survival with return to community) steadily increased from 2006 to 2014 for all three conditions. Regarding the input measure, costs first increased and then decreased during the study period, with a vertex in 2011. Both payments and costs for all three conditions followed the same trend.

S2 Figure a. Survival with return to community rate for three conditions, 2006-2014


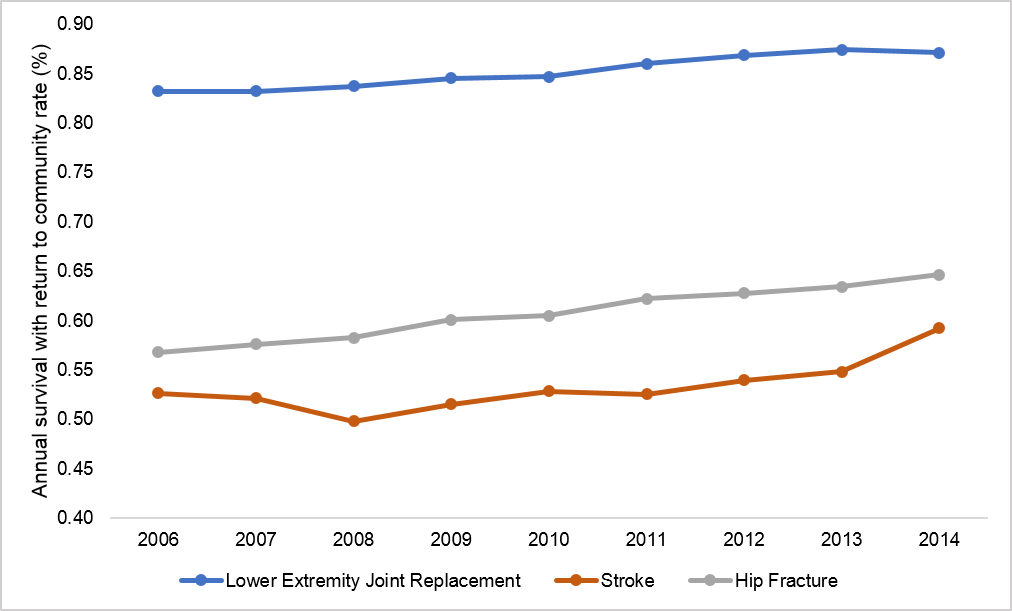


S2 Figure b. Average stay costs per SNF per year for three conditions, 2006-2014


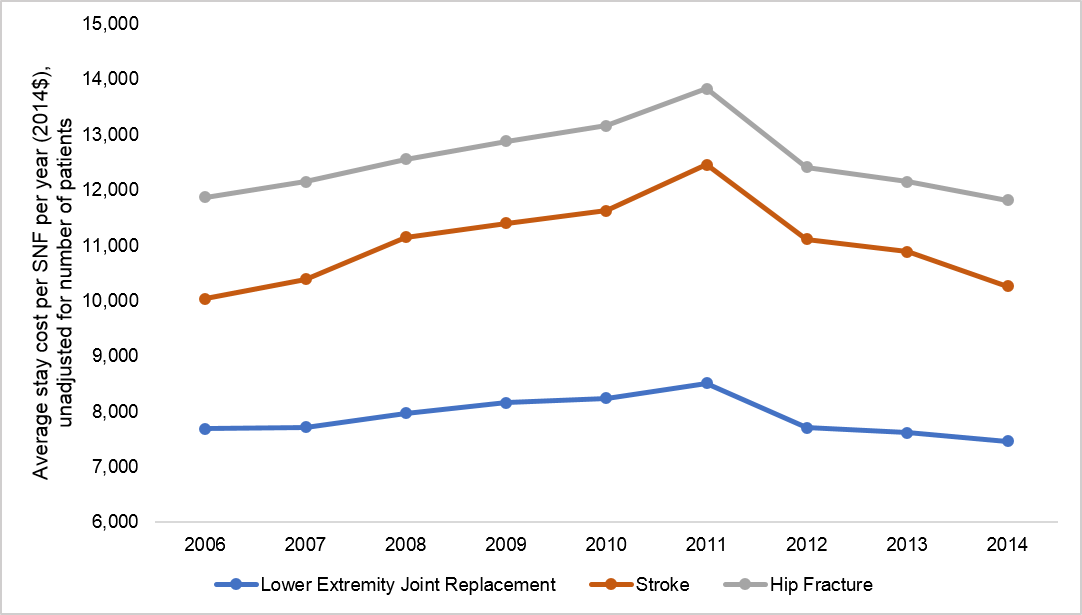

Supplement: S2 Text — (DOCX) [file pone.0215876.s004.docx]
